# β-Carotene Impacts the Liver MicroRNA Profile in a Sex-Specific Manner in Mouse Offspring of Western Diet-Fed Mothers: Results from Microarray Analysis by Direct Hybridization

**DOI:** 10.3390/ijms252312899

**Published:** 2024-11-30

**Authors:** Diana Marisol Abrego-Guandique, Sebastià Galmés, Adrián García-Rodríguez, Roberto Cannataro, Maria Cristina Caroleo, Joan Ribot, Maria Luisa Bonet, Erika Cione

**Affiliations:** 1 Department of Health Sciences, University of Magna Graecia Catanzaro, 88100 Catanzaro, Italy; dianamarisol.abregoguandique@unicz.it (D.M.A.-G.); mariacristina.caroleo@unicz.it (M.C.C.); 2 Laboratory of Molecular Biology, Nutrition, and Biotechnology (LBNB), Nutrigenomics, Biomarkers and Risk Evaluation (NuBE) Research Group, Universitat de les Illes Balears, 07122 Palma, Spain; s.galmes@uib.cat (S.G.); adrian.garcia-rodriguez@uib.cat (A.G.-R.); joan.ribot@uib.es (J.R.); 3 Institut d’Investigació Sanitària Illes Balears (IdISBa), 07120 Palma, Spain; 4 CIBER de Fisiopatología de la Obesidad y Nutrición (CIBEROBN), Instituto de Salud Carlos III, 28029 Madrid, Spain; 5 Galascreen Laboratories, University of Calabria, 87036 Rende, Italy; rcannataro@nutrics.it (R.C.); erika.cione@unical.it (E.C.); 6 Research Division, Dynamical Business & Science Society—DBSS International SAS, Bogotá 110311, Colombia; 7 Artificial Intelligence Research Institute of the Balearic Islands (IAIB), University of the Balearic Islands, 07122 Palma, Spain; 8 Department of Pharmacy, Health and Nutritional Sciences, University of Calabria, 87036 Rende, Italy

**Keywords:** carotenoids, β-carotene, liver, microRNAs, Western diet, lactation, weaning, early-life programming

## Abstract

Maternal unbalanced diets cause adverse metabolic programming and affect the offspring’s liver microRNA (miRNA) profile. The liver is a site of β-carotene (BC) metabolism and a target of BC action. We studied the interaction of maternal Western diet (WD) and early-life BC supplementation on the epigenetic remodeling of offspring’s liver microRNAs. Mouse offspring of WD-fed mothers were given a daily placebo (controls) or BC during suckling. Biometric parameters and liver miRNAome by microarray hybridization were analyzed in newly weaned animals. BC sex-dependently impacted the liver triacylglycerol content. The liver miRNAome was also differently affected in male and female offspring, with no overlap in differentially expressed (DE) miRNAs between sexes and more impact in females. Bioinformatic analysis of DE miRNA predicted target genes revealed enrichment in biological processes/pathways related to metabolic processes, regulation of developmental growth and circadian rhythm, liver homeostasis and metabolism, insulin resistance, and neurodegeneration, among others, with differences between sexes. Fifty-five percent of the overlapping target genes in both sexes identified were targeted by DE miRNAs changed in opposite directions in males and females. The results identify sex-dependent responses of the liver miRNA expression profile to BC supplementation during suckling and may sustain further investigations regarding the long-term impact of early postnatal life BC supplementation on top of an unbalanced maternal diet.

## 1. Introduction

Carotenoids are natural pigments produced by vegetables, algae, and photosynthetic bacteria that play an important role in human health [1]. Among the carotenoids, β-carotene (BC) has been widely studied for its role as a precursor of vitamin A, although it may also exert vitamin A-independent effects [2]. In mammals, vitamin A and BC are crucial for the normal function of vision [3], skin [4], energy and lipid metabolism [2], and neurological processes [5,6], among others. Due to its potential to scavenge free radicals and its interaction with specific cellular signaling pathways, BC has been associated with protective effects against oxidative stress- and inflammation-related diseases, including cardiovascular diseases, type 2 diabetes, obesity, and some types of cancer [7], which are often associated with unbalanced obesogenic diets.

The liver is a central metabolic hub and an important organ for vitamin A and BC metabolism. The liver expresses key BC metabolizing enzymes [8], and BC has recognized hepatoprotective effects in adult animals [9,10]. microRNAs (miRNAs, miR) contribute to the control of hepatic function and metabolism [11,12]. The miRNAs are short non-coding RNA molecules (19–25 nucleotides) that act at the post-transcriptional level to regulate (generally inhibit) gene expression. A connection of BC metabolism with metabolic health through the regulation of specific miRNAs in the liver is suggested by the fact that the ablation of key enzymes in carotenoid metabolism (BCO1 and BCO2) induces hepatic steatosis in mice by altering the farnesoid X receptor/miR-34a/sirtuin 1 axis [13].

Studies in human and animal models indicate that maternal high-fat diet feeding and obesity result in adverse programming of offspring metabolic health at young and adult ages [14]. miRNAs may play a role in metabolic programming by dietary and other factors in early life, serving as epigenetic agents [15]. In particular, animal studies have shown that a maternal high-fat diet during gestation and lactation affects the expression of miRNAs in the liver of fetuses, recently weaned offspring, and adult offspring [16,17,18,19,20]. The impact of BC intake on metabolic programming in early life has been studied little. We previously showed that vitamin A supplementation as BC or retinyl palmitate during suckling differently impacts molecular traits in adipose tissue in rats at weaning, including DNA methylation [21,22]. This suggests potential different effects in the long-term programming of body adiposity. Interestingly, emerging evidence indicates that carotenoids in human milk may play important roles in infant nutrition and development [23], and it is known that breast milk BC content is decreased in obese women [24]. Of note, miRNAs are present at variable levels in breast milk [25,26]. To the best of our knowledge, no previous studies have addressed the interaction of a maternal obesogenic diet and BC supplementation during suckling on the offspring’s liver miRNA expression profile.

**Figure 1:**
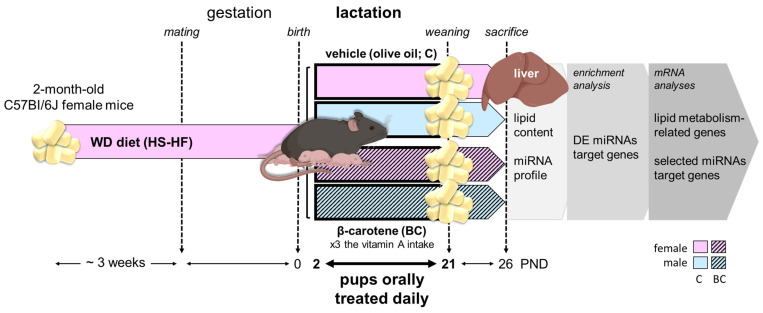
Diagram of the experimental study design. Newborn female and male offspring of WD-fed mothers received β-carotene (BC, dissolved in olive oil), equivalent to ~three-fold the vitamin A ingested daily through maternal milk or vehicle (olive oil; C, control) from postnatal day 2 to 20 and were weaned onto mothers’ diet on day 21 until sacrifice on postnatal day 26 (see Section 4 for further details). WD—Western diet; PND—postnatal day. Images from BioRender.

In this work, we hypothesized that BC supplementation during suckling could modify miRNA expression in the liver of offspring exposed to an unbalanced diet during early development. To test this hypothesis, mouse offspring born to dams fed an obesogenic Western diet (WD) during gestation and lactation were orally supplemented with placebo (controls) or BC during the suckling period. The WD is a purified diet rich in fat and sucrose that emulates common nutritional imbalances in the diet pattern of Westernized societies and is widely used in research on metabolic disturbances and programming. The liver miRNA profile was analyzed in the recently weaned controls and BC animals for differential expression using a microarray direct hybridization method. To investigate potential post-transcriptional regulation by these miRNAs, we generated pathway interaction and miRNA–gene regulatory networks through an integrated analysis of the key miRNAs exhibiting modulation. Figure 1 shows a flowchart of the experimental design used in the study.

## 2. Results

### 2.1. Biometric Parameters

**Figure 2:**
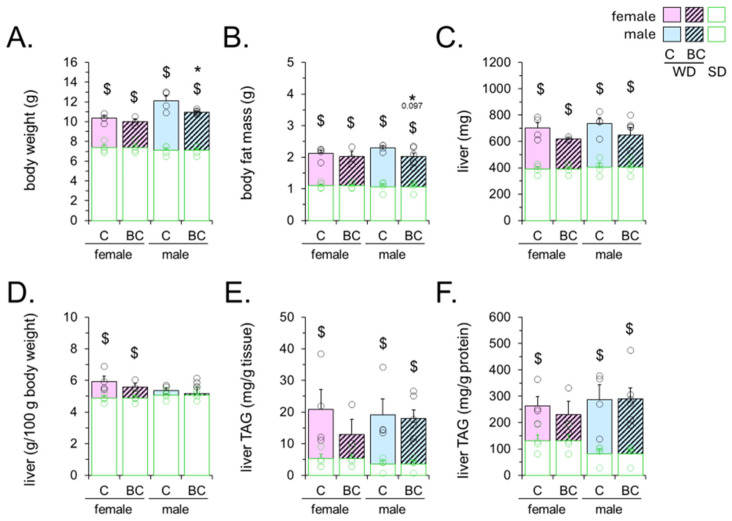
Early-life beta-carotene (BC) effects on biometric parameters of mouse offspring of Western diet (WD)-fed mothers. Body weight (**A**) and total body fat mass ((**B**), acquired with an NMR system) on postnatal day (PND) 24, liver weight (**C**), liver weight as a percentage of body weight (**D**), and liver triacylglycerol (TAG) content per g of tissue (**E**) and per g of protein (**F**) at the end of the experiment. Newborn female and male mice received BC (equivalent to approx. three-fold the vitamin A ingested daily through maternal milk) or vehicle (olive oil; control, C) from PND 2 to 20 and were weaned onto mothers’ diet on PND 21 until sacrifice on PND 26. In green, the corresponding biometric parameters of vehicle-treated male and female offspring born to standard chow-fed mothers (SD), conducted in parallel, are shown. Data are the mean ± SEM of 3–6 animals per group. Circles correspond to individual data. The color of the circles indicates the type of diet: green for SD and black for WD. Statistics (*p* < 0.05, t-Student): $, WD-C or WD-BC vs. SD; *, BC vs. C.

Compared to the chow diet, exposure to a WD rich in saturated fat and sucrose during development led to increased body weight, total body fat mass, liver absolute weight, and liver fat content in the recently weaned offspring of both sexes, as expected (Figure 2). The liver relative weight was increased in the female offspring only (Figure 2D). The male offspring exposed to WD and supplemented with BC showed lower body weight (Figure 2A and Supplementary Table S1) and a tendency towards lower total body fat mass (Figure 2B) compared to the WD-exposed control male offspring. This trend to decreased fat mass reached statistical significance for the retroperitoneal (BC-M = 18.2 ± 0.8 mg, C-M = 23.1 ± 2.8 mg, *p* = 0.027) and the gonadal (BC-M = 102.5 ± 3.1 mg, C-M = 129.2 ± 15.8 mg, *p* = 0.031) white fat depots at sacrifice (Supplementary Table S1). No statistically significant changes in body weight or fat depot mass were observed in the BC-supplemented female offspring. However, the increase in liver fat content brought about by early-life exposure to the WD was selectively attenuated in the BC-supplemented female offspring (Figure 2E,F, showing hepatic triacylglycerol (TAG) levels expressed per gram of fresh tissue and tissue protein, respectively).

### 2.2. Nanostring Analysis of Differentially Expressed miRNAs in the Liver

**Figure 3:**
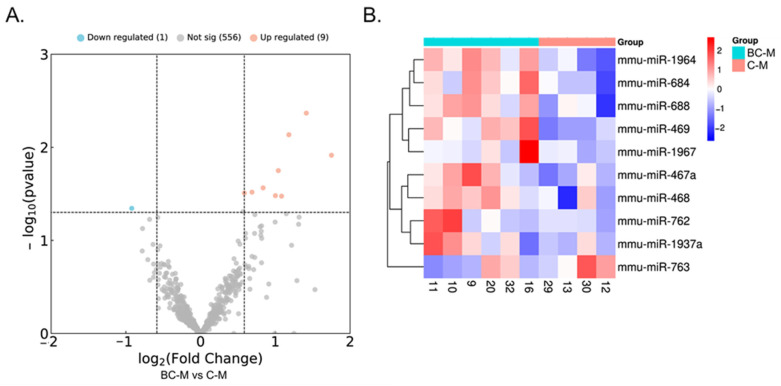
(**A**) Volcano plot and (**B**) heatmap visualizing differentially expressed (DE) miRNAs in the liver of BC and control male mice. In the volcano plot, the blue spots represent downregulated miRNAs, and the pink/red spots upregulated miRNAs with BC. The grey spots represent miRNAs that did not change significantly between the two groups. Cutoff criteria for DE miRNAs were a log2 fold change >0.5 or <−0.5 and *p* < 0.05. C-M, control-male; BC-M, β-carotene-male.

**Figure 4:**
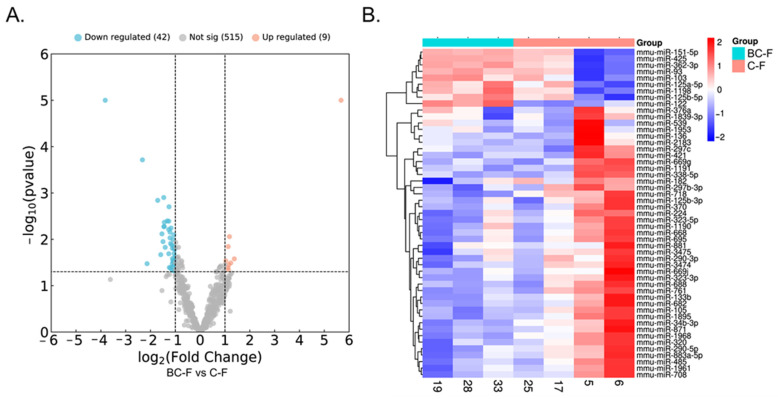
(**A**) Volcano plot and (**B**) heatmap visualizing differentially expressed (DE) miRNAs in the liver of BC and control female mice. In the volcano plot, the blue spots represent downregulated miRNAs, and the pink/red spots upregulated miRNAs with BC. Grey spots represent miRNAs that did not change significantly between the two groups. Cutoff criteria for DE miRNAs were a log2 fold-change >1 or <−1 and *p* < 0.05. C-F, control-female; BC-F, β-carotene-female.

The hepatic miRNAs modulated in the recently weaned, WD-exposed animals by BC supplementation during suckling differed between sexes, both in number and qualitatively. In males, ten miRNAs were differentially expressed (DE) with BC with a log2 fold change >0.5 or <−0.5 and *p* < 0.05; of them, one miRNA was downregulated, and nine were upregulated in the BC-M group compared to the C-M group (Table 1 and Volcano plot in Figure 3A). The expression levels of the DE miRNAs are represented as clustered heatmaps in Figure 3B. In females, 51 miRNAs were DE with a log2 fold change >1 or <−1 and *p* < 0.05; of them, 42 were downregulated, and 9 were upregulated in the BC-F group compared to the C-F group (Table 2, and Volcano plot in Figure 4A). No common DE miRNAs were found in males and females. Figure 4B shows the expression levels of the DE miRNAs in the female animals as heatmaps.

### 2.3. Gene Ontology (GO) Analysis of the Differentially Expressed miRNAs Target Genes

**Figure 5:**
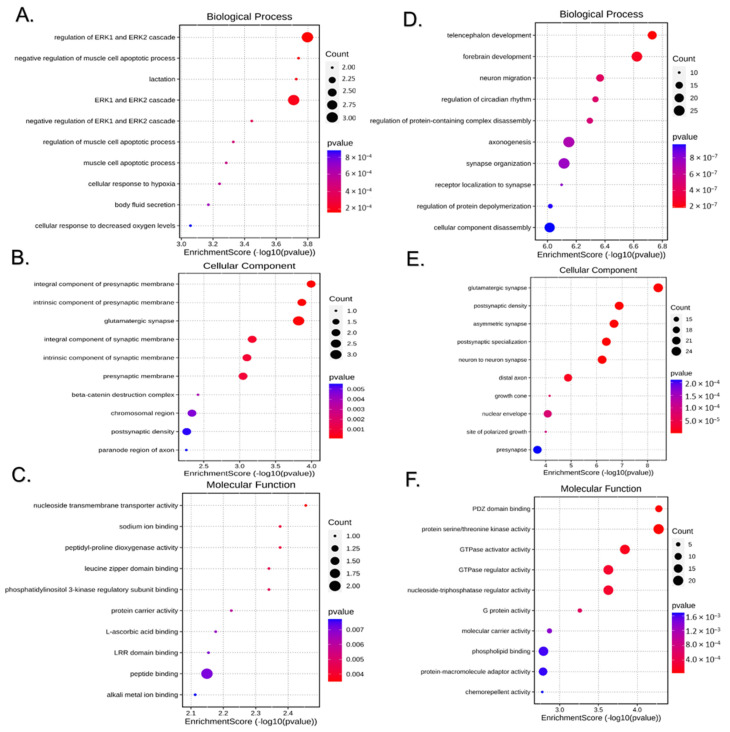
Gene ontology analysis of target genes of miRNAs differentially expressed (DE) in the liver of BC-treated male offspring. Panels (**A**–**C**) (**left**) correspond to the downregulated miRNA targets, and panels (**D**,**F**) (**right**) to the upregulated miRNA targets. The panels show the top 10 most represented biological processes (**A**,**D**), cellular components (**B**,**E**), and molecular functions (**C**,**F**) among the DE miRNA targets. The size of each dot represents the number of input genes associated with the GO term, and the color indicates the *p* value.

**Figure 6:**
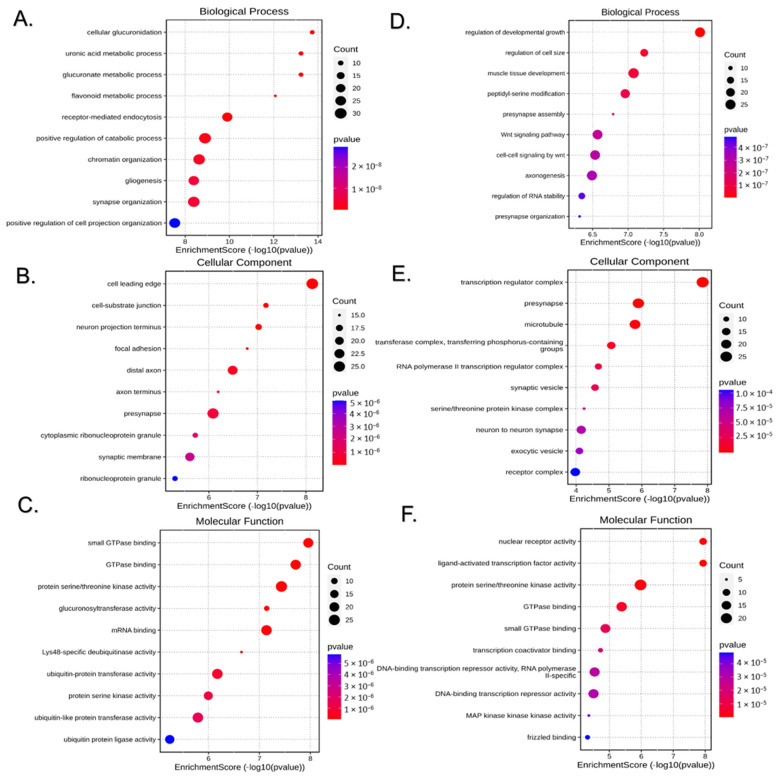
Gene ontology analysis of target genes of miRNAs differentially expressed (DE) in the liver of BC-treated female offspring. Panels (**A**–**C**) (**left**) correspond to the downregulated miRNA targets, and panels (**D**–**F**) (**right**) to the upregulated miRNA targets. The panels show the top 10 most represented biological processes (**A**,**D**), cellular components (**B**,**E**), and molecular functions (**C**,**F**) among the DE miRNA targets. The size of each dot represents the number of input genes associated with the GO term, and the color indicates the *p* value.

The biological significance of the DE miRNAs integrated signature was approached via their target genes. The miRWalk platform predicted 542 target genes for the 10 DE miRNAs in males and 900 target genes for the 51 DE miRNAs in females with a binding probability threshold >0.95. GO enrichment analyses were performed on the respective gene sets for up and downregulated DE miRNAs. GO categories were assigned to the target genes according to the biological processes in which the gene products are involved, the cellular components to which the gene products are located, and their molecular function. Figure 5 shows the top 10 significant results in the three GO categories for the predicted targets of downregulated (Figure 5A–C) and upregulated (Figure 5D,F) DE miRNAs in the liver of BC male offspring. Figure 6 shows the corresponding results for the female offspring. Most of the top enriched terms within the three GO categories varied between sexes, which was expected as the DE miRNAs varied in each sex. Notably, targets of downregulated miRNAs were enriched in processes related to the ERK1 and ERK2 cascade and its regulation in males and to diverse metabolic processes in females (see Figure 5A and Figure 6A). Enriched GO terms perfectly overlapping between male and female offspring were “synapse organization” and “axonogenesis” within biological processes. Additionally, other biological processes and cellular components GO terms related to synapses and neuron biology were found enriched in both sexes, as were molecular function GO terms related to protein serine/threonine kinase activity.

### 2.4. KEGG Pathway Functional Enrichment Analysis

**Figure 7:**
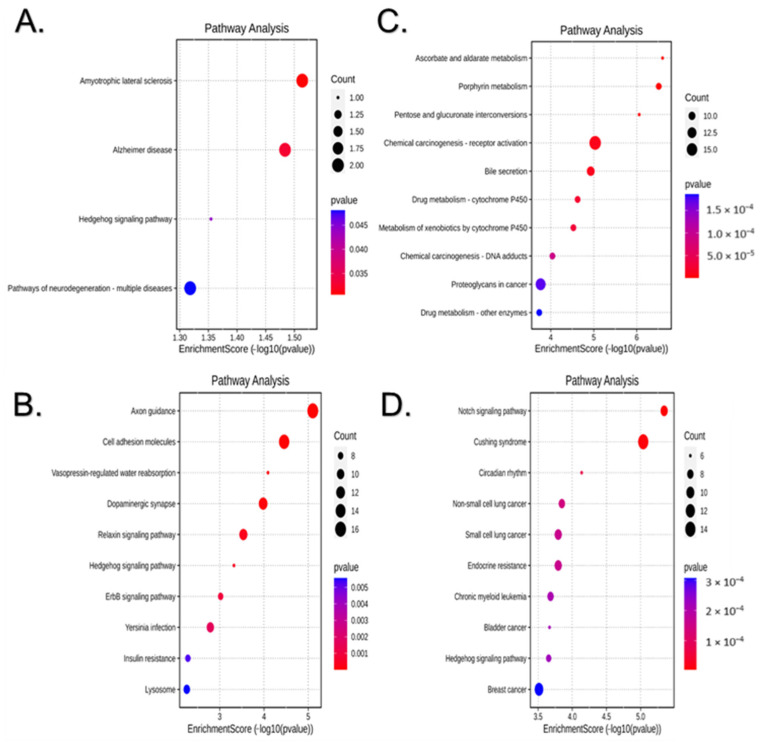
Top KEGG pathways associated with the significantly downregulated (**A**,**C**) and upregulated (**B**,**D**) miRNAs in the liver of BC-supplemented male (**A**,**B**) and female (**C**,**D**) offspring. The size of each dot represents the number of input genes associated with the KEGG pathway, and the color indicates the *p* value.

Figure 7 shows the top KEGG-enriched pathways associated with predicted targets of the DE miRNAs in the liver of BC-supplemented offspring of each sex. Fifty-two pathways were significantly enriched in the BC males (*p*-value < 0.05). Targets of the single downregulated miRNA were associated significantly with four KEGG pathways: Amyotrophic lateral sclerosis, Alzheimer’s disease, pathways of neurodegeneration, and Hedgehog signaling (Figure 7A). The 10 most significantly enriched pathways of targets of the upregulated miRNAs in BC males included cell adhesion molecules, Hedgehog signaling, ErbB signaling, and insulin resistance, among others (Figure 7B). In the BC females, 130 KEGG pathways were significantly enriched. The top 10 enriched pathways associated with the downregulated miRNAs included Ascorbate and aldarate metabolism, Porphyrin metabolism, Pentose and glucuronate interconversions, Chemical carcinogenesis, Bile secretion, and drug metabolism, among others (Figure 7C); additionally, Steroid hormone biosynthesis and retinol metabolism were significantly enriched (*p*-value < 0.05). Finally, the top 10 enriched pathways associated with the upregulated miRNAs in BC females included, among others, Notch signaling, Cushing syndrome, circadian rhythm, non-small/small cell lung cancer, breast cancer, Hedgehog signaling, and Endocrine resistance (Figure 7D).

### 2.5. Construction of miRNA–Gene Interactional Networks, Analysis of Overlapping Target Genes in Both Sexes, and Identification of miRNAs with Bco1 as Target

A comprehensive list of DE miRNA–gene interactions is provided in the Supplementary Materials as an Excel file (with separate sheets for up and downregulated miRNAs in females and males). Based on the miRNA–gene prediction pairs, miRNA–target gene networks were plotted with Cytoscape software v3.9.1 (Supplementary Figure S1). In both sexes, the networks revealed several genes targeted by multiple DE miRNAs. In males, miR-763, the only downregulated miRNA, had a low number of predicted targets, while miR-762 had the highest number, followed by miR-1967, miR-467a-5p, miR-684, miR-468-3p, miR-688, and miR-1964-3p. In females, among the downregulated miRNAs, miR-182-5p had the highest number of targets, followed by miR-320-3p, miR-133b-3p, miR-370-3p, and miR-485-5p, whereas among the upregulated miRNAs in females, miR-93-5p had the highest predicted number of targets, followed by miR-125a-5p, miR-125b-5p, miR-103-3p, miR-122-5p, miR-362-3p, miR-425-5p, and miR-151-5p.

**Figure 8:**
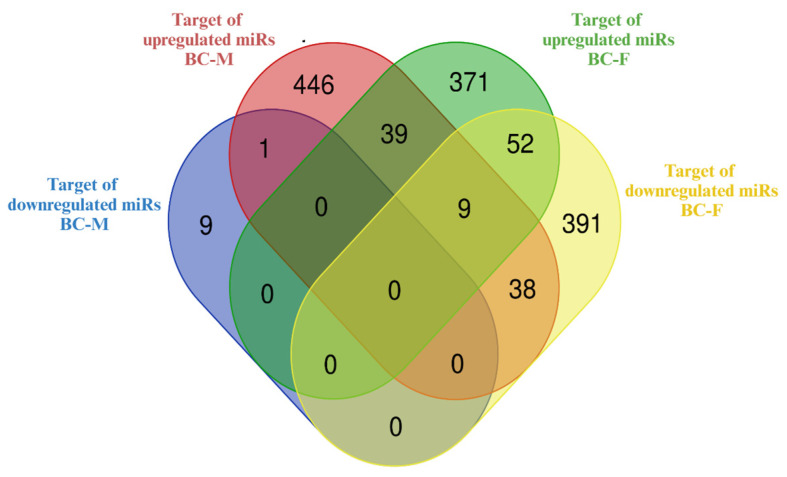
Venn diagram of predicted target genes of the miRNAs differentially expressed in the liver of BC-supplemented males (BC-M) and females (BC-F). Multiple miRNA species, different in the two sexes, target the same genes in both male and female BC-supplemented offspring. Created with Bioinformatics & Evolutionary Genomics.

In an attempt to decipher the major functions of the miRNAs DE in the liver of BC-supplemented offspring, we further focused on predicted targets overlapping in both sexes. It should be noted that even if no DE miRNAs in common were found in the liver of males and females supplemented with BC, it is possible that the target genes of different DE miRNAs partially overlap since the same mRNA can be co-regulated by different miRNAs [27]. Furthermore, the co-regulatory miRNAs might respond differently to a given treatment/intervention. A Venn diagram (Figure 8) showed a total of 86 targets of DE miRNAs overlapping in the two sexes, besides 456 uniquely predicted targets in the male mice, and 814 uniquely predicted targets in the female mice. Twenty-two DE miRNAs—six in males (mmu-miR-762, -684, -467, -1967, -468, -1964) and sixteen in females (mmu-miR-182-5p, -5p, -320-3p, -133b-3p, -485-5p, -668-3p, -224-5p, -323-3p, -708-5p, -1968-5p, -103-3p, -125a-5p, -93-5p, -125b-5p, -362-3p, -122-5p)—were predicted to be involved in the targeting of these 86 overlapping genes. The overlapping genes and their potential regulatory DE miRNAs in the liver of male and female BC-supplemented mice are listed in Supplementary Table S2. Out of the 86 genes that overlapped, 47 were predicted targets of miRNAs that were regulated in opposite directions (up or down) between sexes. KEGG pathway adscriptions of the 86 overlapping targets in both sexes are presented in Supplementary Table S3. It is worth noting that *Pik3r1* was the most involved gene in multiple KEGG pathways (see Table S3).

miRNA-target gene mRNA networks were constructed through Cytoscape considering the 86 overlapping genes and the 22 DE miRNAs potentially involved in their regulation (Supplementary Figure S2). In the BC males, 98 pairs of upregulated miRNAs–downregulated mRNAs were predicted (Figure S2A). Some genes (*Cux1*, *Blcap*, *Zc3h12c*, *Lhx6*, *Nfib*, and *Cadm2*) paired with more than one of the upregulated miRs in BC-M liver, acting at intersections between miRNAs (Figure S2A). In the BC females, 109 miRNA–gene pairs were predicted, of which 51 pairs involved downregulated miRNAs (Figure S2B) and 58 pairs involved upregulated miRNAs (Figure S2C). There were five target genes at the intersections among downregulated miRNAs (*Ubn2*, *Cux1*, *Otud7b*, *Prlr*, and *St8sia2*), and seven target genes at the intersections among upregulated miRNAs (*Etv3*, *Atxn1*, *Trp53inp1*, *Rora*, *Snob1*, *Cacna1b*, and *Kcnk10*). Out of the DE miRNA–gene pairs predicted in male animals, mmu-miR-684, -1967, and -762 are experimentally validated regulatory miRNAs for *Lhx6*, *Nav1*, and *Xpo7*, respectively, whereas out of the pairs predicted in female animals, mmu-miR-125b-5p, -362-3p, -122-5p, and -103-3p are experimentally validated regulatory miRNAs for *Abtb1*, *Atxn1*, *Slc7a1*, and *Pik3r1*, respectively (source miRTarbase).

Besides the systematic bioinformatic approach, a targeted approach was used for *Bco1*, since this gene encodes the crucial enzyme (beta-carotene-15,15′-oxygenase) for the cleavage and metabolism of BC in mammals [28]. Six DE miRNAs were predicted through miRwalk to target the *Bco1* mRNA coding sequence with a binding probability of 1, namely mmu-miR-763 in the male offspring and mmu-miR-668, -105, -370, -323-3p, and -290-3p in the female offspring (Table 3). All these DE miRNAs with *Bco1* as a potential target were downregulated in the liver of offspring born to WD-fed dams following BC supplementation during suckling.

### 2.6. Protein–Protein Interaction Network Analysis

To better understand the interactions among predicted targets of the miRNAs regulated by BC in the liver of young mice born to WD-fed mothers, protein–protein interaction (PPI) networks were constructed using the STRING database. The whole networks are shown in Supplementary Figure S3. Shown in Table 4 are the top 10 hub genes among the 86 overlapping genes potentially regulated by BC supplementation in both sexes, ranked according to their node degree, defined as the number of edges connected to the node. The higher the node degree, the higher the core level of the gene in the network.

### 2.7. Gene Expression Results

**Figure 9:**
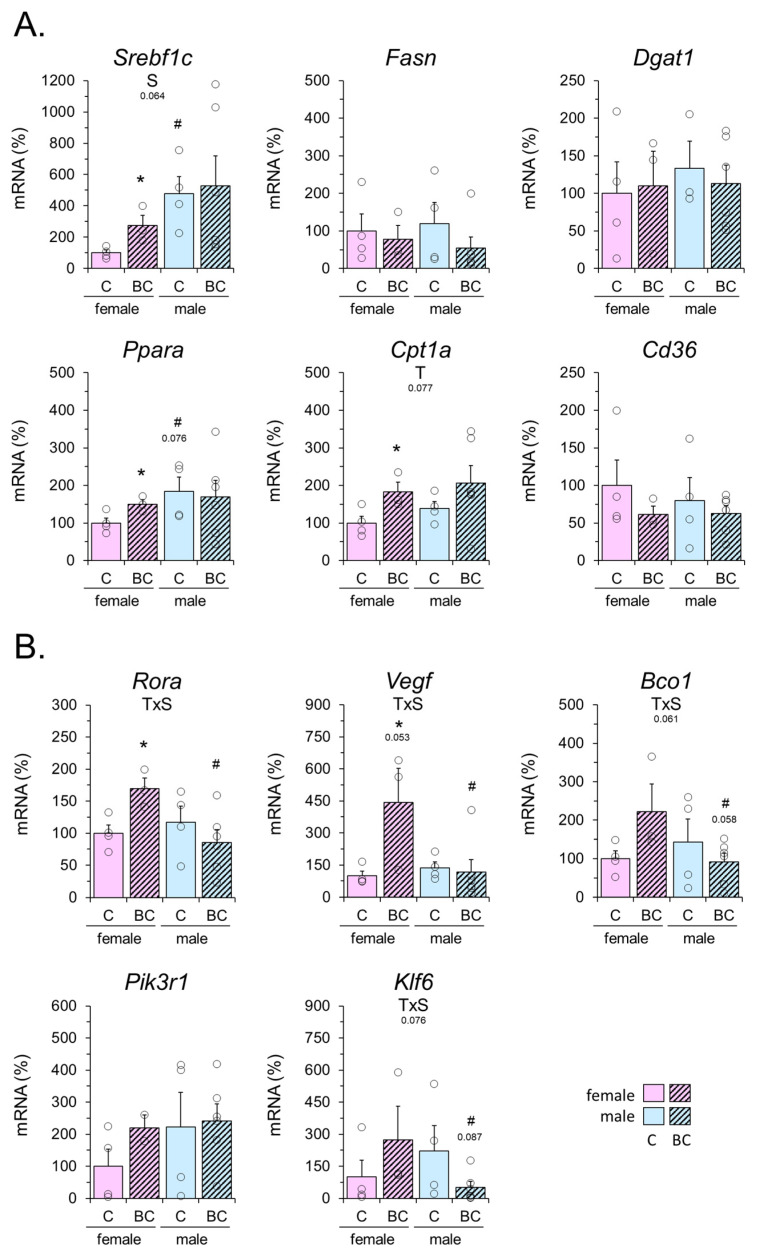
Early-life beta-carotene (BC) treatment effects on liver mRNA levels of the indicated genes related to lipid metabolism (**A**) or selected following miRNA analyses (**B**) in mouse offspring of WD-fed mothers. Newborn female and male mice received BC (equivalent to approx. three-fold the vitamin A ingested daily through maternal milk) or vehicle (olive oil; control) from postnatal day 2 to 20 and were weaned onto WD on day 21 until sacrifice on postnatal day 26. Data are mean ± SEM of 3–6 animals per group and are expressed relative to the mean value of the female control group, which was set to 100. Circles correspond to individual data. Statistics (*p* < 0.05, t-Student): * BC vs. control, # male vs. female. Two-way ANOVA (*p* < 0.05): T, effect of BC treatment; S, effect of sex; TxS, interaction. The *p*-value is indicated when it approaches the significance threshold (0.05 < *p* < 0.1).

To provide context for changes in miRNA profile, we evaluated the hepatic expression of lipid metabolism-related genes in the WD-exposed control and BC-supplemented male and female mice (Figure 9A), prompted by the liver lipid content results. Lipogenesis-related genes (*Srebf1c*, *Fasn*, *Dgat1*), fatty acid oxidation-related genes (*Ppara*, *Cpt1a*), and *Cd36* (whose product is involved in both the cellular and mitochondrial uptake of free fatty acids) were assessed. The mRNA levels of *Srebf1c*, *Ppara*, and *Cpt1a* were significantly upregulated after BC supplementation in the female offspring but not the male offspring, although a trend to *Cpt1a* mRNA upregulation by BC regardless of sex was apparent. *Srebf1c* mRNA levels trended higher in males than females regardless of BC treatment. No noticeable effects were observed for the other lipid metabolism-related genes assessed.

To gain further insight into the possible functional consequences of BC-induced changes in the hepatic miRNAs, we assessed the liver mRNA levels of reported miR-122 target genes (*Klf6*, *Vegf*), miR-122 modulators (*Rora*), the *Pik3r1* gene that was highlighted in our bioinformatic analyses, and the *Bco1* gene (Figure 9B). Significant or nearly significant sex-dependent effects of BC supplementation were found for *Klf6*, *Vegf*, *Rora*, and *Bco1*, as BC supplementation upregulated the expression of these genes in the liver of females but not males. No noticeable effects were observed in *Pik3r1* mRNA levels.

## 3. Discussion

BC is a natural pigment with nutritional functions and interesting bioactive properties in adult animals, including hepatoprotective effects. Maternal unbalanced diets are known to cause mal-programming and affect miRNA expression in the liver of offspring. In this work, the effects of BC supplementation on the top of an unbalanced maternal WD on offspring biometric parameters and hepatic miRNA profile have been studied for the first time to our knowledge. Our results show that BC supplementation had different effects on liver fat accumulation, the liver miRNA profile, and the hepatic expression of lipid metabolism-related genes and predicted targets of differently expressed miRNAs in male and female offspring studied shortly after weaning. No common DE miRNAs were found in male and female offspring, indicating that the response of hepatic miRNA expression patterns to BC supplementation during lactation differs substantially depending on sex. The main results and their possible significance and implications are discussed next.

Identifying sex-dependent responses is crucial to determining new targets, personalizing nutrition and treatment methods, and allowing translation to human health. BC supplementation differentially affected the hepatic miRNA expression profile in male and female animals, with no overlap in DE miRNAs between sexes and a stronger impact in the females. A smaller number of miRNAs responded to BC supplementation in males. Additionally, with the notable exception of miR-122 in females (discussed below), most DE miRNAs changed within a small range in both sexes. The low fold-change observed may be due to upregulated mmu-miR-468-3p in males and mmu-miR-103-3p in females. In the present study, these two miRs were identified in silico as negative regulators of the DROSHA gene, *Dcgr8*, and the efficiency of miRNA processing by DROSHA crucially determines the abundance of miRNAs [29].

The liver is an organ with recognized sexually dimorphic metabolic and cellular responses and is extremely sensitive to the action of sexual hormones [30,31]. Further, the effects of dietary BC on gene expression are also highly organ- and sex-specific [32]. Our results fit this perspective since changes in the hepatic miRNA profile with neonatal BC in WD-exposed offspring strongly differed between sexes. Particularly noteworthy was the sex-dependent response to early-life BC supplementation of hepatic miR-122 expression, markedly upregulated (×51-fold) in the female offspring and unaffected in the male offspring. miR-122 is the predominant liver miRNA, making up 70% of the total miRNA population [33]. Notably, miR-122 has been described in rats as a female-predominant miRNA related to nutritional conditions [34]. miR-122 is a critical regulator of hepatic homeostasis and lipid metabolism [35]. Mice lacking miR-122 develop metabolically dysregulated conditions in the liver (hepatosteatosis) and hepatocellular carcinoma [36]. However, the role of miR-122 in regulating lipid metabolism appears to be complex, and seemingly contradictory results have been reported. In particular, there are reports sustaining miR-122 inhibits hepatic lipogenesis (e.g., [37,38,39]) and reports sustaining miR-122 promotes hepatic lipogenesis (e.g., [40,41]). In this work, we found a reduced accumulation of liver TAG accompanying the upregulation of hepatic miR-122 levels in group BC-F compared with C-F following early-life exposure to a WD. The reduced hepatic TAG accumulation in the BC females was linked to significantly higher expression levels of the fatty acid oxidation-related genes *Ppara* and *Cpt1a*, but also of the gene for the lipogenic transcription factor SREBP1c compared with control females. Although none of the lipid metabolism-related genes analyzed are validated targets of miR-122, we note that the significant gene expression differences versus sex-matched controls observed in the BC females were not present in the BC males, who lacked the induction of miR-122 and presented similar liver TAG levels than controls after exposure to the WD. Interestingly, a maternal high-fat diet leads to decreased miR-122 expression and increased miR-370 expression and fat content in the liver of recently weaned mouse offspring [18,42]. These changes are opposite to those triggered in our experiment by BC supplementation during suckling in the female offspring of WD-fed dams, namely increased miR-122 and decreased miR-370 and liver TAG content (Table 2 and Figure 1). The reduced miR-370 aligns with the increased *Cpt1a* mRNA levels in the liver of the BC-F group, as miR-370 acts on the 3′ UTR of *Cpt1a* mRNA and reduces the expression of this enzyme, which is crucial for fatty acid β oxidation [43].

Even though BC supplementation could regulate these genes in a sex-dependent manner independently of its effects on miRNAs, it is remarkable that both hepatic miR-122 levels and the mRNA levels of validated or proposed miR-122 target genes such as *Klf6* and *Vegf* were sex-dependently affected by the BC treatment. Intriguingly, however, the expression of *Klf6* and *Vegf* was upregulated rather than downregulated in the liver of BC females overexpressing miR-122. The *Klf6* gene is a strongly validated target of murine miR-122 according to miRTarBase; its protein product, Krüppel-like factor 6, is a transcription factor pro-fibrogenic in the liver but also a tumor suppressor [44]. The *Vegf* gene was described as a miR-122 target [45], though it does not appear as a validated one in the miRTarBase. It should be noted that our *Vegf* gene expression results might align with a previous report that the *Vegf* protein product—vascular endothelial growth factor, key for angiogenesis—is induced in cultured endothelial cells and in vivo in skeletal muscle by a miR-122 mimic [46].

We approached the biological significance of changes elicited by early-life BC supplementation on the hepatic miRNA profile by GO annotation and KEGG enrichment analysis of the DE miRNAs predicted targets. In both sexes, the Hedgehog signaling pathway, which regulates hepatic lipid metabolism and its zonation [47], was identified as one of the top enriched KEGG pathways affected. Significant GO terms retrieved in male animals indicated additional biological processes potentially relevant to maintaining liver homeostasis, such as the ERK1/ERK2 cascade [48]. KEGG pathways selectively enriched in the BC males included neurodegenerations/Alzheimer’s disease and insulin resistance, two processes related to each other [49] and potentially to BC nutrition and metabolism. The protective effects of BC against Alzheimer’s disease have been described [5,6], and serum BC levels are reduced in patients with this disease [50,51]. Strikingly, the strongest genetic risk factor for Alzheimer’s disease [52], the *APOE4* genotype, may also condition a relatively low dietary BC bioavailability [53]. This is suggested by the fact that, following a BC diet, engineered mice expressing human *APOE4* had lower plasma and tissue BC levels (due to higher hepatic but not intestinal *Bco1* expression) than mice expressing human *APOE3* [53]. A connection between insulin resistance and BC, in turn, may arise from BC effects on low-density lipoprotein (LDL) oxidation. LDL is the main carrier of circulating BC [54], and there is evidence that BC can inhibit the oxidative modification of LDL [55]. High oxidized LDL levels are associated with insulin resistance [56], metabolic syndrome [57], and atherosclerosis [58]. Oxidized LDL is also neurotoxic and has been related to Alzheimer’s disease [59], even if this latter condition has a multifactorial etiology. It is well established that similar to humans, male mice are less insulin sensitive and more susceptible to unhealthy diet-induced metabolic disorders than females [60,61,62]. Therefore, epigenetic modulation of these health endpoints by neonatal BC supplementation through effects on miRNAs could be particularly interesting in males. Contrary to findings in males, neurodegenerations/Alzheimer’s disease and insulin resistance were not among the top enriched KEGG pathways in the females; instead, enrichment in drug metabolism and cancer-related pathways was revealed.

Overall, supplementing BC during the suckling period impacts the hepatic expression of miRNAs in recently weaned mice exposed to an unfavorable diet during development, and bioinformatic analyses indicate that the affected miRNAs may target genes involved in clinically relevant pathways known to be modulated in a beneficial manner by BC/vitamin A in adult animals. We suggest that changes in these miRNAs could represent early epigenetic mediators of long-term effects of early-life postnatal BC supplementation in offspring exposed to an unbalanced maternal diet during development.

Six DE miRNAs in males and sixteen DE miRNAs in females were predicted through in silico analyses to regulate 86 overlapping genes across both sexes. Of these, forty-seven genes (55% percent) were targeted by miRNAs with opposite (down or up) regulation between sexes. One of those genes was *Rora*, encoding the retinoic acid receptor-related orphan receptor alpha transcription factor (RORα). *Rora* is a circadian rhythm gene [63]— an enriched pathway in our GO analysis in males and KEGG analysis in females— and is involved in the transcriptional control of metabolism. Sex-dependent differences in the expression of the *Rora* gene in the liver during aging have been described [64]. Interestingly, the expression of miR-122—a miRNA with a marked sex-dependent response in our experiment—is induced in the liver by free fatty acids in a RORα-dependent manner, and this is associated with suppression of lipogenesis and TAG levels in the liver (and also in remote skeletal muscle tissue) [65]. Further, an agonist of RORα attenuates nonalcoholic fatty liver progression in mice via upregulation of miR-122 [66]. Our gene expression results showed that, in recently weaned mice exposed to WD during development, neonatal BC supplementation was associated with increased *Rora* mRNA levels in the supplemented female mice but not in males, in which levels were rather decreased. Opposite effects of BC supplementation on *Rora* expression in female and male animals align with opposite regulation by BC of miRNAs predicted to target *Rora*, namely miR-370 downregulated in the BC females and miR-1967 upregulated in the BC males (Supplementary Table S2). The induction of *Rora* expression in the BC females could contribute to and is consistent with the selective induction of miR-122 and the lower liver TAG content in this group, in light of previous reports [65,66].

Bioinformatics analysis of protein–protein interactions between predicted targets of miRNAs differentially expressed upon BC supplementation revealed many connections involving *Pik3r1*. This gene was a predicted target of DE miR-762 in males and of miR-320-3p and miR-103-3p in females and is a validated target of the latter miR (Table S2). *Pik3r1* encodes a regulatory subunit of phosphoinositide 3-kinases (PI3K). PI3K are crucial components of the PI3K/AKT/mTOR pathway regulating cell growth, metabolism, survival, apoptosis, and autophagy [67]. The *Pik3r1* protein product binds, stabilizes, and inhibits the PI3K catalytic subunit, thus exerting a complex regulatory role. It modulates the metabolic actions of insulin, and its mutation has been associated with insulin resistance [68]. The *Pik3r1* protein product also has tumor suppressor potential since the downregulation of its levels favors the constitutive activation of downstream Akt signaling, which can induce carcinogenesis [69]. The microarray results and bioinformatic analysis suggested that BC supplementation during suckling could modulate this regulatory hub via miRNAs in offspring exposed to adverse dietary conditions during development. This cannot be discarded, although we did not detect significant differences in the hepatic expression of *Pik3r1* at the mRNA level among the experimental groups.

Interestingly, while none of the upregulated DE miRNAs targeted *Bco1*, up to five downregulated DE miRNAs in females were predicted to target this gene. In males, only the single downregulated miRNA targeted *Bco1*. This scenario suggested that the hepatic expression of *Bco1* could be increased following oral BC supplementation during suckling, particularly in the female offspring. In good concordance, the gene expression results pointed to sex-dependent effects of BC supplementation on the hepatic expression of *Bco1*, with increased mRNA levels with BC supplementation selectively in the BC females. Dietary BC conversion to vitamin A retinoids through BCO1 decreases adiposity [70,71], circulating cholesterol [72], and atherosclerosis progression [73,74] in various experimental animal models. Additionally, emerging evidence suggests that BCO1 activity may counteract the development of hepatic steatosis and have other beneficial health effects independent of its role in carotenoid cleavage [13,75]. Therefore, increased hepatic *Bco1* mRNA levels, as observed here in the BC-F, may be considered a positive outcome consistent with the liver TAG results. Interestingly, BCO1 was recently described to inhibit the metastatic potential of cancer neuroblastoma cells by regulating differentiation-related miRNAs [75]. In this context, it is to be noted that our KEGG analysis suggested the targeting of cancer-related pathways by DE miRNAs with BC selectively in the female offspring.

This is the first report to evaluate the impact of neonatal BC supplementation in the context of an obesogenic maternal diet on the miRNA profile in the offspring’s liver. BC-induced changes in the hepatic miRNA profile observed in the recently weaned animals might be relevant for contemporary hepatic and systemic metabolism and also for the programming of offspring’s health in later life. Our study provides a global vision that BC supplementation in early life can impact tissue miRNA expression in a sex-dependent manner and that sexual differences in metabolic programming could rely on miRNAs, among other factors. The present study has some limitations that should be noted. First, our results are based on a mouse model with a small sample size and are limited to the short term. An in-depth investigation should be carried out to ascertain the long-term programming impact of observed effects in recently weaned animals and the translatability of findings to humans. Second, the current study is largely based on bioinformatics analysis of microarray data; further cell and animal experiments should be designed to explore the specific physiological functions of miRNAs affected by BC supplementation in early life and their downstream targets in the regulation of liver metabolism and development, as well as the ultimate mechanisms of the BC-induced sex-dependent regulation of these miRNAs revealed in this work.

## 4. Materials and Methods

### 4.1. Experimental Design

The animal protocol of this study was reviewed and approved by the Animal Experimentation Ethics Committee (CEEA) of the University of the Balearic Islands (#ECC/566/2015, of March 20). Institutional use and care guidelines for laboratory animals were followed. C57BI/6J female mice kept under constant conditions of 12/12 h light–dark cycle at room temperature of 22 °C and with free access to food and water were used. The female mice were switched from the standard chow diet (LASQCdiet^®^Rod14-H, Lage, Germany) to a WD pellet diet (D12079B, Research Diets, New Brunswick, NJ, USA) three weeks before mating with males of the same strain fed the standard chow. The dams continued to be fed the WD during the whole gestation and lactation period. From postnatal day (PND) 2 until weaning (on PND21), the pups were orally treated daily, with the aid of a pipette, with 10 μL olive oil (control group) or the same volume of β-carotene (BC group) dissolved in olive oil. The amount of supplemented BC was increased progressively from 10 μg BC per pup per day on PND2 to 18 μg on PND20 and corresponded to approx. three-fold the vitamin A ingested daily through maternal milk. The daily amount of BC to be administered was calculated from the reported vitamin A concentration as retinyl ester in mouse milk [76], the estimated volume of daily milk intake of mouse pups (which increases along the suckling period) [77], and the vitamin A equivalency of BC (VEB) in oil. We used a VEB of 2.5:1, which is based on the reported micrograms of BC in oil required to form 1 μg retinol in children, as in previous work with a similar animal study design [21]. At weaning, offspring were separated by sex, making up four experimental groups: control male and female mice (C-M and C-F, respectively) and BC-supplemented male and female mice (BC-M and BC-F), with *n* = 3 to 6 animals per group, coming from at least three different WD-fed mothers. The animals continued to be fed with WD until sacrifice on PND26. On PND24, body composition was analyzed using an Echo MRI-700 body composition analyzer (Echo Medical Systems LLC, Houston, TX, USA). At sacrifice, the liver and fat depots were dissected, weighed, frozen immediately in liquid nitrogen, and stored at −80 °C. Biometric parameters and liver protein and fat content were also obtained for vehicle-treated male and female offspring born to standard chow-fed mothers weaned onto standard chow, raised in parallel.

### 4.2. Liver Total Protein and Triacylglycerol Content

Approximately 20 mg of liver were weighed and homogenized with PBS (1/20 dilution) in ice. For total protein determination, the homogenate was centrifuged (10,000× *g*, 1 min, at room temperature). The resulting supernatant was used for the protein quantification following the PierceTM BCA Protein Assay Kit (Thermo Scientific, Rockford, IL, USA) protocol. The liver triacylglycerol (TAG) content was quantified using the QCA colorimetric kit (Química Clínica Aplicada S.A., Tarragona, Spain).

### 4.3. miRNA Isolation and Quantification

The mirVana^TM^ miRNA Isolation Kit (Ambion by Life Technologies, Grand Island, NY, USA) was used to extract miRNA from liver samples following the manufacturer’s instructions. First, 0.024 ± 0.005 g of liver samples were homogenized in 250 μL of lysis binding buffer, on ice. A spike-in of *Arabidopsis thaliana* microRNA (ath-miR-159a, 1 ƿmol, 2 μL) was added to each sample as a standard, vortexed for 30 s, and incubated on ice for 5 min. Then, 1/10 volume of miRNA Homogenate Additive (55 μL) was added to the homogenate, mixed by vortexing, and left for 10 min on ice. Subsequently, 250 μL phenol/chloroform was added and vortexed for 1 min. The mixture was then centrifuged for 10 min at 10,000× *g* at 4 °C to separate the aqueous phase from the organic phase. The aqueous phase (upper) was pipetted and transferred to an RNase-free Eppendorf (200 μL) and 1.25 volumes of 100% ethanol were added at room temperature and mixed by inversion (5 times). Each sample was passed through the columns in aliquots of 250 μL and centrifuged for 30 s at 10,000× *g*, the eluate was discarded. This was repeated until all the lysate/ethanol mixture was passed through the filter. The filter/column was washed with 700 μL of miRNA wash solution 1 and centrifuged for 10 s at 10,000× *g*, and then the column was washed with 500 μL of wash buffer solution 2/3, centrifuged twice at 10,000× *g* for 10 s, and the eluate was discarded each time. A spin of 1 min was performed to remove the remains of the filter. The columns were transferred to a new collection RNase-free tube, and 50 μL of RNase-free water at 95 °C were added and incubated for 2 min. Finally, it was centrifuged for 30 s at maximum speed, and the RNA eluate was collected and stored at −80 °C. Nucleic acids extracted were quantified by spectrophotometry with NanoDrop ND-1000 (ThermoFisher Scientific, Waltham, MA, USA) at 260 nm, using 2 µL, which allows it to be quantified in ng/µL with reproducibility and precision. Samples with nucleic acid concentration >100 ng/μL were used. RNA quality and integrity were checked by the 260/280 ratio and confirmed by 1% agarose gel electrophoresis.

### 4.4. miRNA Assessment

In total, 150 ng of liver RNA/miRNA was used as input for miRNA profiling using the n-counter flex NanoString Technology (Seattle, WA, USA). Mature miRNAs underwent an annealing procedure for 13 min with the following conditions: 94 °C for 1 min, 65 °C for 2 min, and 45 °C for 10 min. Then, miRNAs were ligated to a species-specific tag sequence (miRtag) via a thermally controlled splinted ligation at 48 °C for 3 min, 47 °C for 3 min, 46 °C for 3 min, 45 °C for 5 min, and 65 °C for 10 min (24 min in total). The unligated miRtags were removed by enzymatic purification using Ligase cleanup^TM^ enzyme for 70 min. miRtagged mature miRNAs were then hybridized with the mouse miRNA V 1.5 panel (LBL-C0068-02) according to the instructions at nCounter^®^ miRNA Expression Assay User Manual (NanoString Technologies, Seattle, WA, USA) for 22 h at 65 °C. The unhybridized CodeSet was removed with automated purification performed on the nCounter Prep Station, and the remaining target probe complexes were transferred and bound to an imaging surface as previously described [78]. The data output was imported into nSolver™ 4.0 Analysis software (NanoString Technologies, Seattle, WA, USA). Raw data were exported as an Excel table for further bioinformatics analysis. The n-counter flex NanoString Technology used for miRNA profiling is widely recognized for its high analytical performance and was shown to provide results comparable to qPCR [79,80]. Further, comprehensive analyses of miRNA expression profiles by microarray and next-generation sequencing (RNA-seq) gave similar results [81].

### 4.5. Bioinformatic Analysis

Volcano plots were created using SRPLOT tools based on the normalized counts to identify significant DE miRNAs. The R heatmap function (v1.0.12) was used to perform heatmap clustering. miRwalk—which integrates predictions from multiple miRNA target prediction databases, including TargetScan, and miRBD—was used to identify predicted miRNA target genes, applying a binding probability threshold >0.95. The miRNA–target genes networks were plotted with Cytoscape software v3.9.1 (Cytoscape Team, San Diego, CA, USA), which overlaps genes from the three aforementioned databases. Finally, gene ontology (GO) and Kyoto Encyclopedia of Genes and Genomes (KEGG) analysis of the predicted targets of the differentially expressed miRNAs in BC-M and BC-F groups were conducted using DIANA miRPath tools [82]. The STRING online tool was used to identify protein–protein interactions among identified key genes. STRING provides direct (physical) interactions and indirect (functional) associations that stem from computational prediction, knowledge transfers between organisms, and interactions aggregated from other (primary) databases. The ability of the DE miRNAs to interact with the *Bco1* mRNA 3′-UTR and CDS (coding sequence) was evaluated using the miRwalk platform, applying a binding probability of 1, which indicates a high-quality prediction. Experimentally validated microRNA-target interactions were assessed using miRTarbase.

### 4.6. mRNA Analyses

Total liver RNA was isolated using phenol-based TriPure Reagent (Roche Diagnostics GmbH, Mannheim, Germany). Once isolated, RNA integrity was checked by 1% agarose gel electrophoresis stained with SYBR Safe (ThermoFisher Scientific, Waltham, MA, USA), and the isolated RNA quantification and quality were assessed using the NanoDrop ND-1000 spectrophotometer (Nano-Drop Technologies, Wilmington, DE, USA). Isolated RNA was reverse transcribed to complementary DNA (cDNA) using the highly sensitive cDNA synthesis kit (iScript cDNA, Bio-Rad Laboratories, Madrid, Spain) and an Applied Biosystems 2720 Thermal Cycler (Applied Biosystems, Madrid, Spain). Gene expression was measured by specific cDNA amplification and semi-quantification following the StepOnePlus protocol (Applied Biosystems, Madrid, Spain) and using the Power SYBR Green PCR Master Mix (Applied Biosystems, Madrid, Spain). All primers used were purchased from Sigma-Aldrich (Sigma-Aldrich S.A., Madrid, Spain), and the sequences are the following for Forward (F, 5′-3′) and Reverse (R, 3′-5′):

*Bco1* (F: GAGCAAGTACAACCATTGGT; R: AACTCAGACACCACGATTC);
*Cd36* (F: GTGGCAAAGAACAGCAGCAA; R: CCAACAGACAGTGAAGGCTCA);
*Cpt1a* (F: GCTCGCACATTACAAGGACAT; R: TGGACACCACATAGAGGCAG);
*Dgat1* (F: TGGCCTGCCCCATGCGTGAT; R: ACCCACTGCCAGGCGCTTCT);
*Fasn* (F: CGGCGAGTCTATGCCACTAT; R: ACACAGGGACCGAGTAATGC);
*Klf6* (F: GGACCAAATTCATTCTAGCTCGGG; R: AGGCGTCGCCATTACCCTTG); *Pik3r1*(F: GAAGTTGCTCTACCCAGTGTCC; R: CGATAGCCGTTCTTTTCATTTGGAT); *Ppara* (F: CGTTTGTGGCTGTGCAAGTT; R: AGAGAGGACAGATGGGGCTC);
*Rora* (F: CAATGCCACCTACTCCTGTCC; R: GCCAGGCATTTCTGCAGC);
*Srebf1c* (F: CAGCGGTTTTGAACGACA; R: GCCAGAGAAGCAGAAGAGAAG);
*Vegf* (F: CACGACAGAAGGAGAGCAGA; R: ATCAGCGGCACACAGGAC).

Data were normalized against 18S ribosomal RNA gene expression.

### 4.7. Statistical Analysis

Comparisons between the vehicle-treated (control group, C) and β-carotene (BC)-treated groups were carried out for each sex (C-M/BC-M and C-F/BC-F). For miRNA expression profiling, the DESeq2 R package [83] was used to perform data normalization based on miRNA raw counts and the analysis of differentially expressed (DE) miRNA between sex-matched BC and C groups. miRNAs that showed a log2 fold change >0.5 or <−0.5 in BC-M vs. C-M and >1 or <−1 in BC-F vs. C-F with a *p*-value <0.05 were used as cutoffs to filter DE miRNAs. Similarly, a cutoff of *p* < 0.05 was used for significant enrichment results in GO and KEGG analyses. The top 10 enriched terms were plotted with dotplot using the clusterProfiler R package.

For biometric and gene expression (mRNA) analyses, data are shown as means and standard error of the mean (SEM). Differences among the groups based on the BC-treatment (T), sex (S) and interaction effects (TxS) between these two factors on the dependent variable were assessed by two-way ANOVA. A two-tailed Student’s t test was used for binary comparisons. The significance threshold for all statistics was set at *p* < 0.05. *p* values between 0.05 and 0.10 were considered nonsignificant tendencies. Analyses were performed with SPSS for Windows (SPSS version 27.0, Chicago, IL, USA).

## Figures and Tables

**Table 1:** Differentially expressed miRNAs in the liver of males with BC supplementation *.

miRNA	Fold Change	Regulation	*p*-Value
mmu-miR-763	−1.89363	Down	0.0453
mmu-miR-1937a/b	3.366737	Up	0.0121
mmu-miR-762	2.665820	Up	0.004291
mmu-miR-468	2.267680	Up	0.007326
mmu-miR-1967	2.11883	Up	0.033513
mmu-miR-469	2.056269	Up	0.017799
mmu-miR-688	2.001758	Up	0.033073
mmu-miR-684	1.785582	Up	0.027278
mmu-miR-1964	1.610750	Up	0.030326
mmu-miR-467a	1.500623	Up	0.030996

* Cutoff criteria for DE miRNAs were a log2 fold change >0.5 or <−0.5 and *p* < 0.05.

**Table 2:** Differentially expressed miRNAs in the liver of females with BC supplementation *.

miRNA	Fold Change	Regulation	*p*-Value
mmu-miR-1191	−14.1521	Down	3.09 × 10^−7^
mmu-miR-2183	−5.01241	Down	0.000193
mmu-miR-376a	−4.40317	Down	0.033524
mmu-miR-1968	−3.26993	Down	0.001442
mmu-miR-539	−3.00592	Down	0.021402
mmu-miR-136	−2.90541	Down	0.007614
mmu-miR-669g	−2.8221	Down	0.011202
mmu-miR-338-5p	−2.76262	Down	0.001253
mmu-miR-682	−2.75437	Down	0.005321
mmu-miR-323-5p	−2.72259	Down	0.005302
mmu-miR-290-5p	−2.7219	Down	0.004294
mmu-miR-182	−2.65349	Down	0.014895
mmu-miR-320	−2.50812	Down	0.004008
mmu-miR-761	−2.40692	Down	0.00612
mmu-miR-871	−2.40115	Down	0.004071
mmu-miR-1839-3p	−2.40041	Down	0.020481
mmu-miR-34b-3p	−2.38487	Down	0.001983
mmu-miR-881	−2.36257	Down	0.040452
mmu-miR-224	−2.32138	Down	0.009298
mmu-miR-1961	−2.31858	Down	0.007012
mmu-miR-125b-3p	−2.2945	Down	0.041596
mmu-miR-297c	−2.28392	Down	0.012285
mmu-miR-421	−2.25396	Down	0.005722
mmu-miR-883a-5p	−2.21618	Down	0.013146
mmu-miR-3474	−2.19501	Down	0.04617
mmu-miR-3475	−2.17952	Down	0.033841
mmu-miR-297b-3p	−2.16784	Down	0.015613
mmu-miR-1953	−2.16066	Down	0.025989
mmu-miR-695	−2.1502	Down	0.039103
mmu-miR-323-3p	−2.13416	Down	0.028574
mmu-miR-133b	−2.13306	Down	0.010116
mmu-miR-370	−2.12883	Down	0.03029
mmu-miR-1895	−2.08625	Down	0.007972
mmu-miR-688	−2.07886	Down	0.047323
mmu-miR-485	−2.0763	Down	0.014138
mmu-miR-290-3p	−2.07055	Down	0.020309
mmu-miR-105	−2.06476	Down	0.015803
mmu-miR-669j	−2.06456	Down	0.035964
mmu-miR-1190	−2.0396	Down	0.048281
mmu-miR-708	−2.03612	Down	0.016461
mmu-miR-668	−2.03022	Down	0.029321
mmu-miR-718	−2.01753	Down	0.024074
mmu-miR-122	51.125	Up	1.74 × 10^−25^
mmu-miR-103	2.598296	Up	0.026379
mmu-miR-125b-5p	2.352268	Up	0.032295
mmu-miR-362-3p	2.265612	Up	0.008745
mmu-miR-151-5p	2.229765	Up	0.035369
mmu-miR-125a-5p	2.19763	Up	0.044985
mmu-miR-1198	2.186721	Up	0.014346
mmu-miR-93	2.170918	Up	0.041012
mmu-miR-425	2.050926	Up	0.029188

* Cutoff criteria for DE miRNAs with BC were a log2 fold-change >1 or <−1 and *p* < 0.05.

**Table 3:** Differentially expressed miRNAs in the liver of BC-supplemented offspring potentially regulating *Bco1*.

Gene	Sex	miRNAs Downregulated	miRNAs Upregulated
*Bco1*	Male	mmu-miR-763	-
	Female	mmu-miR-668, -105, -370, -323-3p, -290-3p	-

**Table 4:** Top 10 genes in the protein–protein interaction networks involving the overlapping targets in both sexes of miRNAs expressed differentially in the liver following BC supplementation, ranked by node degree.

Males Upregulated		Females Downregulated		Females Upregulated	
Genes	Node Degree	Genes	Node Degree	Genes	Node Degree
*Pik3r1*	23	*Pik3r1*	29	*Pik3r1*	23
*Arrb1*	16	*Arrb1*	16	*Atxn1*	19
*Kalrn*	15	*Xpo7*	16	*Btrc*	13
*Atxn1*	13	*Kalrn*	13	*Suv39h1*	12
*Cacna1b*	9	*Cacna1b*	11	*Eif4a2*	10
*Ube2l3*	9	*Celf1*	11	*Erc1*	9
*Xpo7*	9	*Foxp2*	11	*Rora*	8
*Btrc*	8	*Cux1*	9	*Ube2l3*	7
*Dab2ip*	8	*Adam22*	6	*Cacna1b*	6
*Ndel1*	8	*Cntn2*	6	*Dgcr8*	6

## Data Availability

The raw data supporting the conclusions of this article will be made available by the authors on reasonable request.

## Supplementary Materials

The following supporting information can be downloaded at https://www.mdpi.com/article/10.3390/ijms252312899/s1.
