# Supplementary material for: β-Carotene Impacts the Liver MicroRNA Profile in a Sex-Specific Manner in Mouse Offspring of Western Diet-Fed Mothers: Results from Microarray Analysis by Direct Hybridization
Source: Int J Mol Sci. 2024 Nov 30;25(23):12899. doi: 10.3390/ijms252312899 (PMC11641259; doi:10.3390/ijms252312899)
Supplement: Supplementary file 1 [file ijms-25-12899-s001.zip › a. ijms-3198844 supplementary material.pdf]

International Journal of *Molecular Sciences*

MDPI

Article

β-carotene impacts the liver microRNA profile in a sex-specific manner in mouse offspring of Western diet-fed mothers: results from microarray analysis by direct hybridization

Diana Marisol Abrego-Guandique [1,4,§], Sebastià Galmés [2,6,7,§], Adrián García-Rodríguez [2,6,7], Roberto Cannataro [4,5], Maria Cristina Caroleo [1,4], Joan Ribot [2,6,7], M. Luisa Bonet [2,6,7,8,*], Erika Cione [3,4]

[1] Department of Health Sciences, University of Magna Graecia Catanzaro, 88100 Catanzaro, Italy. dianamarisol.abregoguandique@unicz.it; mariacristina.caroleo@unicz.it
[2] Laboratory of Molecular Biology, Nutrition, and Biotechnology (LBNB); Nutrigenomics, Biomarkers and Risk Evaluation (NuBE) research group, Universitat de les Illes Balears, 07122 Palma, Spain. luisabonet@uib.es; joan.ribot@uib.es; s.galmes@uib.cat; adrian.garcia-rodriguez@uib.cat
[3] Department of Pharmacy, Health and Nutritional Sciences, University of Calabria, 87036 Rende, Italy. erika.cione@unical.it
[4] Galascreen Laboratories, University of Calabria, 87036 Rende, Italy. rcannataro@nutrics.it
[5] Research Division, Dynamical Business & Science Society – DBSS International SAS, Bogotá 110311, Colombia. rcannataro@nutrics.it
[6] Institut d'Investigació Sanitària Illes Balears (IdISBa), 07120 Palma, Spain. luisabonet@uib.es; joan.ribot@uib.es; s.galmes@uib.cat; adrian.garcia-rodriguez@uib.cat
[7] CIBER de Fisiopatología de la Obesidad y Nutrición (CIBEROBN), Instituto de Salud Carlos III, 28029 Madrid, Spain. luisabonet@uib.es; joan.ribot@uib.es; s.galmes@uib.cat; adrian.garcia-rodriguez@uib.cat
[8] Artificial Intelligence Research Institute of the Balearic Islands (IAIB), University of the Balearic Islands, 07122 Palma, Spain. luisabonet@uib.es

* Correspondence: luisabonet@uib.es
§ Equally contributed

International Journal of
Molecular Sciences

MDPI

Supplementary Materials

Table S1. Biometric parameters at sacrifice

	CONTROL		BC	
	**FEMALE**	**MALE**	**FEMALE**	**MALE**
**Body weight (g)**	11.9±0.37	13.7±0.53	11.1±0.35	12.5±0.17*
**BAT weight (mg)**	74.1±5.63	63.7±9.04	59.7±4.56	65.9±4.04
**Inguinal WAT weight (mg)**	167±5.15	149±14.27	138±19.94	125±9.30
**Retroperitoneal WAT weight (mg)**	27.8±2.82	23.1±2.77	18.3±1.58	18.2±0.83*
**Gonadal WAT weight (mg)**	59.1±2.30	129±15.8	59.2±10.9	102±3.06*
**VAT/SAT ratio**[a]	0.52±0.04	1.03±0.07	0.59±0.15	0.99±0.07

The offspring of Western diet-fed mouse dams was orally supplemented with placebo (control group) or beta-carotene (BC) from day 2 to day 21 of life and sacrificed at day 26 when tissues were sampled. BAT, brown adipose tissue; WAT, white adipose tissue; VAT, visceral adipose tissue; SAT, subcutaneous adipose tissue; [a] ratio between VAT (gonadal plus retroperitoneal) and SAT (inguinal) depots mass. *, $p<0.05$, BC vs CONTROL, Student's *t*-test.

International Journal of Molecular Sciences

MDPI

Table S2. Overlapping target genes in both sexes of miRNAs differentially expressed with neonatal beta-carotene (BC) supplementation in the liver of mouse offspring of Western diet-fed mothers and the DE miRNAs related to their regulation

	MALE MICE		FEMALE MICE	
**Gene**	**miRNAs**	**Regulation by BC**	**miRNAs**	**Regulation by BC**
**Abhd13**	mmu-miR-468-3p	Up	mmu-miR-320-3p	Down
**Abtb1**	mmu-miR-762	Up	**mmu-miR-125b-5p***	Up
**Adam22**	mmu-miR-1967	Up	mmu-miR-182-5p	Down
**Adamtsl3**	mmu-miR-762	Up	mmu-miR-485-5p	Down
**Agap1**	mmu-miR-684	Up	mmu-miR-320-3p	Down
**Arrb1**	mmu-miR-762	Up	mmu-miR-133b-3p	Down
**Asxl3**	mmu-miR-684	Up	mmu-miR-182-5p<br>mmu-miR-362-3p	Down<br>Up
**Atxn1**	mmu-miR-684	Up	mmu-miR-103-3p<br>mmu-miR-125a-5p<br>**mmu-miR-362-3p***<br>mmu-miR-93-5p	Down<br>Down<br>Down<br>Down
**Blcap**	mmu-miR-467a-5p<br>mmu-miR-762	Up<br>Up	mmu-miR-320-3p	Down
**Bmerb1**	mmu-miR-762	Up	mmu-miR-362-3p	Up
**Btbd7**	mmu-miR-467a-5p	Up	mmu-miR-93-5p	Up
**Btrc**	mmu-miR-762	Up	mmu-miR-103-3p	Up
**Cacna1b**	mmu-miR-762	Up	mmu-miR-133b-3p<br>mmu-miR-125a-5p<br>mmu-miR-125b-5p	Down<br>Up<br>Up
**Cadm2**	mmu-miR-468-3p<br>mmu-miR-684	Up<br>Up	mmu-miR-93-5p	Up
**Celf1**	mmu-miR-762	Up	mmu-miR-133b-3p	Down
**Cers6**	mmu-miR-1967	Up	mmu-miR-320-3p	Down
**Cntn2**	mmu-miR-762	Up	mmu-miR-668-3p	Down
**Cplx3**	mmu-miR-762	Up	mmu-miR-1968-5p	Down
**Csnk1g1**	mmu-miR-762	Up	mmu-miR-708-5p<br>mmu-miR-93-5p	Down<br>Up
**Cux1**	mmu-miR-1967<br>mmu-miR-467a-5p<br>mmu-miR-684	Up<br>Up<br>Up	mmu-miR-133b-3p<br>mmu-miR-320-3p	Down<br>Down
**Dab2ip**	mmu-miR-762	Up	mmu-miR-182-5p	Down
**Dazap2**	mmu-miR-1967	Up	mmu-miR-125a-5p	Up
**Dcun1d1**	mmu-miR-1967	Up	mmu-miR-182-5p	Down
**Dgcr8**	mmu-miR-468-3p	Up	mmu-miR-103-3p	Up
**Dync1li2**	mmu-miR-467a-5p	Up	mmu-miR-182-5p<br>mmu-miR-103-3p	Down<br>Up
**Egflam**	mmu-miR-468-3p	Up	mmu-miR-103-3p	Up
**Eif4a2**	mmu-miR-762	Up	mmu-miR-103-3p	Up
**Eif5a2**	mmu-miR-467a-5p	Up	mmu-miR-125a-5p	Up
**Emc10**	mmu-miR-762	Up	mmu-miR-485-5p	Down
**Enah**	mmu-miR-684	Up	mmu-miR-224-5p	Down
**Erc1**	mmu-miR-762	Up	mmu-miR-93-5p	Up
**Etv3**	mmu-miR-1964-3p	Up	mmu-miR-103-3p	Up
**Fam168a**	mmu-miR-684	Up	mmu-miR-182-5p	Down
**Fbxo41**	mmu-miR-762	Up	mmu-miR-182-5p	Down
**Foxp2**	mmu-miR-762	Up	mmu-miR-182-5p	Down
**Frmd8**	mmu-miR-762	Up	mmu-miR-370-3p	Down

International Journal of Molecular Sciences

**Fzd3**	mmu-miR-762	Up	mmu-miR-320-3p	Down
**Kalrn**	mmu-miR-684	Up	mmu-miR-323-3p	Down
**Kcnk10**	mmu-miR-762	Up	mmu-miR-125a-5p<br>mmu-miR-125b-5p<br>mmu-miR-182-5p	Up<br>Up<br>Down
**Kpna1**	mmu-miR-684	Up	mmu-miR-103-3p	Up
**Lhx6**	mmu-miR-467a-5p<br>**mmu-miR-684***	Up<br>Up	mmu-miR-93-5p	Up
**Lin28b**	mmu-miR-467a-5p	Up	mmu-miR-103-3p	Up
**Mfap3l**	mmu-miR-468-3p	Up	mmu-miR-93-5p	Up
**Mkrn1**	mmu-miR-467a-5p	Up	mmu-miR-93-5p	Up
**Myrf**	mmu-miR-467a-5p	Up	mmu-miR-133b-3p	Down
**Nav1**	**mmu-miR-1967***	Up	mmu-miR-103-3p	Up
**Ndel1**	mmu-miR-762	Up	mmu-miR-103-3p	Up
**Nfib**	mmu-miR-467a-5p<br>mmu-miR-684	Up<br>Up	mmu-miR-125a-5p	Up
**Osbpl6**	mmu-miR-468-3p	Up	mmu-miR-103-3p	Up
**Otud7b**	mmu-miR-762	Up	mmu-miR-133b-3p<br>mmu-miR-320-3p	Down<br>Down
**Pbx1**	mmu-miR-1967	Up	mmu-miR-125a-5p	Up
**Pik3r1**	mmu-miR-762	Up	mmu-miR-320-3p<br>**mmu-miR-103-3p***	Down<br>Up
**Plagl1**	mmu-miR-1967	Up	mmu-miR-125a-5p	Up
**Ppp1r16b**	mmu-miR-762	Up	mmu-miR-320-3p	Down
**Prdm16**	mmu-miR-467a-5p	Up	mmu-miR-182-5p	Down
**Prlr**	mmu-miR-1967	Up	mmu-miR-182-5p<br>mmu-miR-485-5p	Down<br>Down
**Prr14l**	mmu-miR-762	Up	mmu-miR-93-5p	Up
**Rad18**	mmu-miR-467a-5p	Up	mmu-miR-320-3p	Down
**Retreg3**	mmu-miR-1967	Up	mmu-miR-125a-5p	Up
**Rora**	mmu-miR-1967	Up	mmu-miR-103-3p<br>mmu-miR-125b-5p<br>mmu-miR-370-3p	Up<br>Up<br>Down
**Runx1t1**	mmu-miR-1967	Up	mmu-miR-133b-3p	Down
**Sbno1**	mmu-miR-1967	Up	mmu-miR-103-3p<br>mmu-miR-125a-5p<br>mmu-miR-125b-5p	Up<br>Up<br>Up
**Slc6a17**	mmu-miR-762	Up	mmu-miR-125a-5p<br>mmu-miR-125b-5p	Up<br>Up
**Slc7a1**	mmu-miR-762	Up	**mmu-miR-122-5p***	Up
**Slc8a1**	mmu-miR-762	Up	mmu-miR-103-3p<br>mmu-miR-362-3p	Up<br>Up
**Sntb2**	mmu-miR-1967	Up	mmu-miR-93-5p	Up
**Soga1**	mmu-miR-762	Up	mmu-miR-320-3p	Down
**St8sia2**	mmu-miR-468-3p	Up	mmu-miR-485-5p	Down
**Stard13**	mmu-miR-684	Up	mmu-miR-182-5p	Down
**Stum**	mmu-miR-762	Up	mmu-miR-668-3p	Down
**Suv39h1**	mmu-miR-467a-5p	Up	mmu-miR-125a-5p	Up
**Taf9b**	mmu-miR-467a-5p	Up	mmu-miR-125b-5p	Up
**Tnrc6b**	mmu-miR-467a-5p	Up	mmu-miR-362-3p	Up
**Trp53inp1**	mmu-miR-468-3p	Up	mmu-miR-93-5p<br>mmu-miR-125b-5p	Up<br>Up
**Trps1**	mmu-miR-684	Up	mmu-miR-125a-5p	Up
**Tspan18**	mmu-miR-762	Up	mmu-miR-485-5p	Down
**Ube2l3**	mmu-miR-762	Up	mmu-miR-125b-5p	Up

International Journal of
Molecular Sciences
MDPI

**Ubn2**	mmu-miR-684	Up	mmu-miR-224-5p	Down
			mmu-miR-320-3p	Down
**Ubxn2b**	mmu-miR-468-3p	Up	mmu-miR-93-5p	Up
**Usf3**	mmu-miR-684	Up	mmu-miR-122-5p	Up
**Xpo7**	**mmu-miR-762***	Up	mmu-miR-668-3p	Down
**Zbtb37**	mmu-miR-467a-5p	Up	mmu-miR-182-5p	Down
**Zc3h12c**	mmu-miR-467a-5p	Up	mmu-miR-93-5p	Up
	mmu-miR-468-3p	Up	mmu-miR-103-3p	Up
**Zfp174**	mmu-miR-762	Up	mmu-miR-224-5p	Down
**Zfp704**	mmu-miR-684	Up	mmu-miR-182-5p	Down
**Znrf3**	mmu-miR-467a-5p	Up	mmu-miR-125a-5p	Up
			mmu-miR-133b-3p	Down

*Bold highlight indicates the miRNAs validated for the gene.

International Journal of Molecular Sciences

MDPI

Table S3. KEGG pathway adscriptions of overlapping target genes in both sexes of miRNAs differentially expressed with neonatal beta-carotene supplementation in the liver of mouse offspring of Western diet-fed mothers

FEMALE MICE- target-miRNA downregulated		
**ID pathway**	**Pathways**	**Gene**
mmu05207	Chemical carcinogenesis - receptor activation	Cacna1b, Pik3r1, Arrb1,
mmu05205	Proteoglycans in cancer	Pik3r1, Fdz3
mmu04010	MAPK signaling pathway	Cacna1b, Arrb1
mmu04810	Regulation of actin cytoskeleton	Enah,
mmu04360	Axon guidance	Enah, Pik3r1, Fdz3,
mmu05211	Renal cell carcinoma	Pik3r1
mmu04015	Rap1 signaling pathway	Enah,Pik3r1
mmu05223	Non-small cell lung cancer	Pik3r1
mmu04062	Chemokine signaling pathway	Pik3r1, Arrb1
mmu04510	Focal adhesion	Pik3r1
mmu04071	Sphingolipid signaling pathway	Cers6, Pik3r1
mmu04550	Signaling pathways regulating pluripotency of stem cells	Pik3r1, Fdz3
mmu04666	Fc gamma R-mediated phagocytosis	Pik3r1
mmu05218	Melanoma	Pik3r1
mmu04722	Neurotrophin signaling pathway	Pik3r1
mmu05212	Pancreatic cancer	Pik3r1
mmu05220	Chronic myeloid leukemia	Pik3r1
mmu04144	Endocytosis	Agap1, Arrb1
mmu04068	FoxO signaling pathway	Pik3r1
mmu04012	ErbB signaling pathway	Pik3r1
mmu04211	Longevity regulating pathway	Pik3r1
mmu05225	Hepatocellular carcinoma	Pik3r1, fdz3
mmu04670	Leukocyte transendothelial migration	Pik3r1
mmu05226	Gastric cancer	Pik3r1, fdz3
mmu05221	Acute myeloid leukemia	Pik3r1, Runx1t1
mmu04070	Phosphatidylinositol signaling system	Pik3r1
mmu04611	Platelet activation	Pik3r1
mmu05417	Lipid and atherosclerosis	Pik3r1
mmu04390	Hippo signaling pathway	Fzd3
mmu04917	Prolactin signaling pathway	Prlr, Pik3r1
mmu04024	cAMP signaling pathway	Pik3r1
mmu04660	T cell receptor signaling pathway	Pik3r1
mmu04934	Cushing syndrome	Fdz3
mmu01521	EGFR tyrosine kinase inhibitor resistance	Pik3r1
mmu00600	Sphingolipid metabolism	Cers6
mmu05017	Spinocerebellar ataxia	Pik3r1, Rora
mmu04923	Regulation of lipolysis in adipocytes	Pik3r1
mmu04140	Autophagy - animal	Pik3r1
mmu04340	Hedgehog signaling pathway	Csnk1g1, Arrb1
mmu05213	Endometrial cancer	Pik3r1
mmu05224	Breast cancer	Pik3r1, Fdz3
mmu03013	Nucleocytoplasmic transport	Xpo7
mmu04935	Growth hormone synthesis, secretion and action	Pik3r1
mmu05210	Colorectal cancer	Pik3r1
mmu04213	Longevity regulating pathway - multiple species	Pik3r1
**FEMALE MICE- target-miRNA upregulated**		
mmu04330	Notch signaling pathway	Atxn1,
mmu04710	Circadian rhythm	Rora, Btrc

International Journal of Molecular Sciences

MDPI

mmu05223	Non-small cell lung cancer	Pik3r1
mmu01522	Endocrine resistance	Pik3r1
mmu05222	Small cell lung cancer	Pik3r1
mmu05220	Chronic myeloid leukemia	Pik3r1
mmu04340	Hedgehog signaling pathway	Btrc
mmu05224	Breast cancer	Pik3r1
mmu05207	Chemical carcinogenesis - receptor activation	Cacna1b, Kpna1, Pik3r1
mmu04218	Cellular senescence	Btrc, Pik3r1
mmu04668	TNF signaling pathway	Pik3r1
mmu04140	Autophagy - animal	Pik3r1
mmu04917	Prolactin signaling pathway	Pik3r1
mmu05214	Glioma	Pik3r1
mmu04310	Wnt signaling pathway	Btrc, Znf3
mmu05212	Pancreatic cancer	Pik3r1
mmu05215	Prostate cancer	Pik3r1
mmu05169	Epstein-Barr virus infection	Pik3r1
mmu05226	Gastric cancer	Pik3r1
mmu04152	AMPK signaling pathway	Pik3r1
mmu05165	Human papillomavirus infection	Pik3r1
mmu04390	Hippo signaling pathway	Btrc
mmu05206	MicroRNAs in cancer	Pik3r1
mmu05161	Hepatitis B	Pik3r1
mmu05160	Hepatitis C	Pik3r1
mmu04550	Signaling pathways regulating pluripotency of stem cells	Pik3r1
mmu05167	Kaposi sarcoma-associated herpesvirus infection	Pik3r1
mmu05410	Hypertrophic cardiomyopathy	Slc8a1, Pik3r1
mmu05225	Hepatocellular carcinoma	Pik3r1
mmu05218	Melanoma	Pik3r1
mmu05412	Arrhythmogenic right ventricular cardiomyopathy	Slc8a1
mmu04370	VEGF signaling pathway	Pik3r1
mmu04010	MAPK signaling pathway	Cacna1b
mmu04066	HIF-1 signaling pathway	Pik3r1
mmu05022	Pathways of neurodegeneration - multiple diseases	Cacna1b, Atxn1
mmu05162	Measles	Pik3r1
mmu05211	Renal cell carcinoma	Pik3r1
mmu05414	Dilated cardiomyopathy	Slc8a1
mmu04071	Sphingolipid signaling pathway	Pik3r1
mmu04068	FoxO signaling pathway	Pik3r1
mmu04978	Mineral absorption	Slc8a1
mmu05017	Spinocerebellar ataxia	Atxn1, Rora, Pik3r1
mmu04935	Growth hormone synthesis, secretion and action	Pik3r1
mmu05210	Colorectal cancer	Pik3r1
mmu04360	Axon guidance	Pik3r1
mmu04961	Endocrine and other factor-regulated calcium reabsorption	Slc8a1
mmu04261	Adrenergic signaling in cardiomyocytes	Slc8a1,
mmu04722	Neurotrophin signaling pathway	Pik3r1
mmu05163	Human cytomegalovirus infection	Pik3r1
**MALE MICE- target-miRNA upregulated**		
mmu04360	Axon guidance	Enah, Pik3r1, Fdz3
mmu04514	Cell adhesion molecules	Cntn2
mmu04728	Dopaminergic synapse	Cacna1b, arrb1
mmu04926	Relaxin signaling pathway	Pik3r1, arrb1
mmu04340	Hedgehog signaling pathway	Btrc, arrb1
mmu04012	ErbB signaling pathway	Pik3r1

International Journal of Molecular Sciences MDPI

mmu04931	Insulin resistance	Pik3r1
mmu05170	Human immunodeficiency virus 1 infection	Btrc, Pik3r1
mmu04725	Cholinergic synapse	Cacna1b, Pik3r1
mmu04015	Rap1 signaling pathway	Enah, Pik3r1
mmu05032	Morphine addiction	Cacna1b, Arrb1
mmu04670	Leukocyte transendothelial migration	Pik3r1
mmu05132	Salmonella infection	Kpna1, Dync1li2
mmu04710	Circadian rhythm	Rora, Btrc
mmu00600	Sphingolipid metabolism	Cers6
mmu04010	MAPK signaling pathway	Cacna1b, arrb1
mmu04660	T cell receptor signaling pathway	Pik3r1
mmu05142	Chagas disease	Pik3r1
mmu04151	PI3K-Akt signaling pathway	Prlr, Pik3r1
mmu05165	Human papillomavirus infection	Pik3r1
mmu04928	Parathyroid hormone synthesis, secretion and action	Arrb1
mmu04371	Apelin signaling pathway	Slc8a1,
mmu04668	TNF signaling pathway	Pik3r1, Dab2ip
mmu04727	GABAergic synapse	Cacna1b
mmu05207	Chemical carcinogenesis - receptor activation	Cacna1b, Pik3r1, Kpna1, Arrb1
mmu04935	Growth hormone synthesis, secretion and action	Pik3r1
mmu04810	Regulation of actin cytoskeleton	Enah, pik3r1
mmu01522	Endocrine resistance	pik3r1
mmu05418	Fluid shear stress and atherosclerosis	pik3r1,
mmu04723	Retrograde endocannabinoid signaling	Cacna1b
mmu04722	Neurotrophin signaling pathway	Pik3r1
mmu04014	Ras signaling pathway	Pik3r1
mmu04930	Type II diabetes mellitus	Cacna1b, Pik3r1
mmu04071	Sphingolipid signaling pathway	Cers6, Pik3r1,
mmu05231	Choline metabolism in cancer	Pik3r1
mmu04917	Prolactin signaling pathway	Prlr, Pik3r1
mmu04721	Synaptic vesicle cycle	Cacna1b
mmu04978	Mineral absorption	Slc8a1
mmu04915	Estrogen signaling pathway	Pik3r1
mmu05160	Hepatitis C	Pik3r1

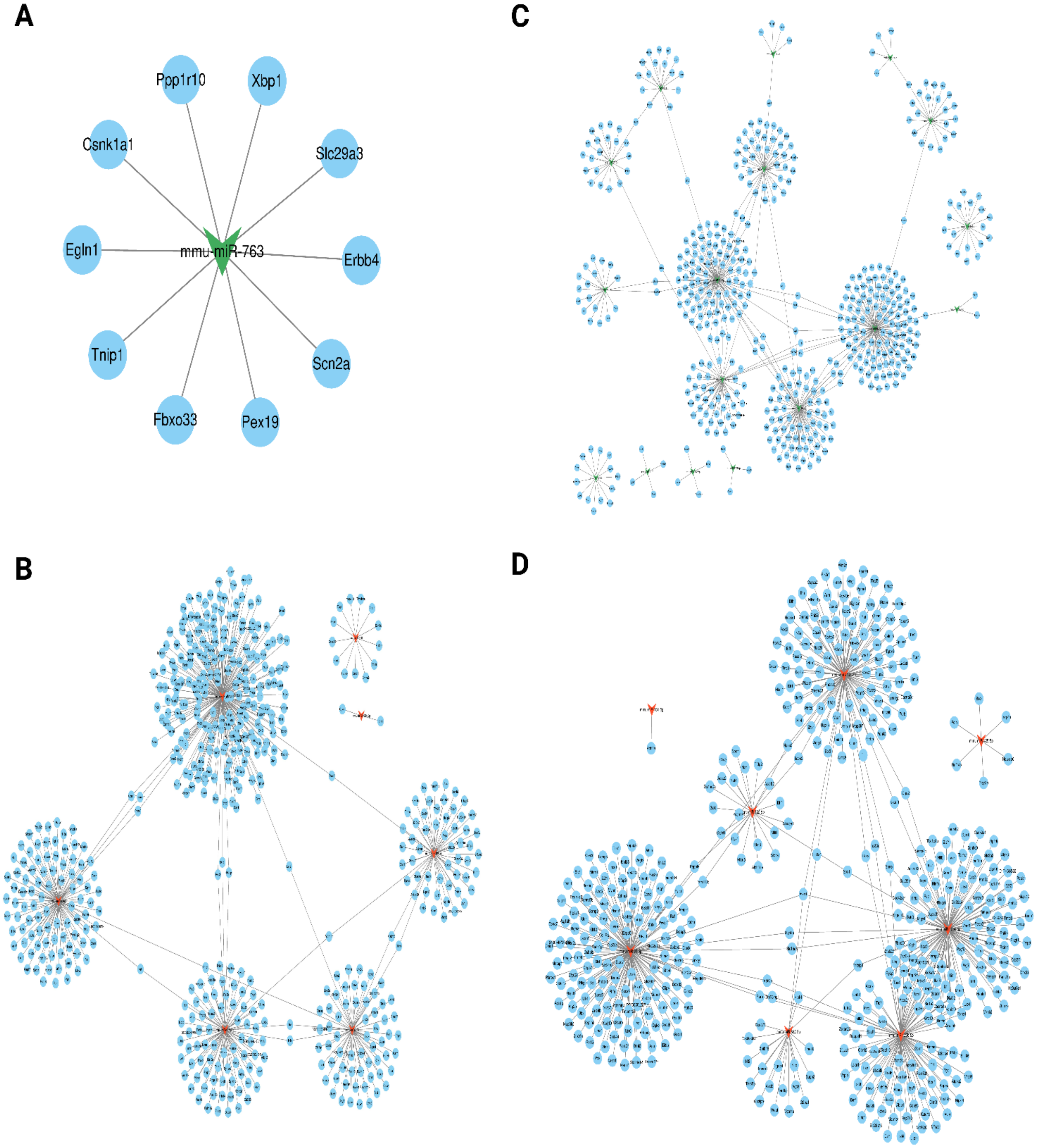

Figure S1. miRNA-gene interaction networks of miRNAs differentially expressed with neonatal beta-carotene (BC) supplementation in the liver of mice born from Western diet-fed dams and their predicted gene targets. Each individual node represents a miRNA or gene. Networks are for A) The single miRNA downregulated in the BC males, B) The upregulated miRNAs in the BC males, C) The downregulated miRNAs in BC females, and D) The upregulated miRNAs in the BC females.

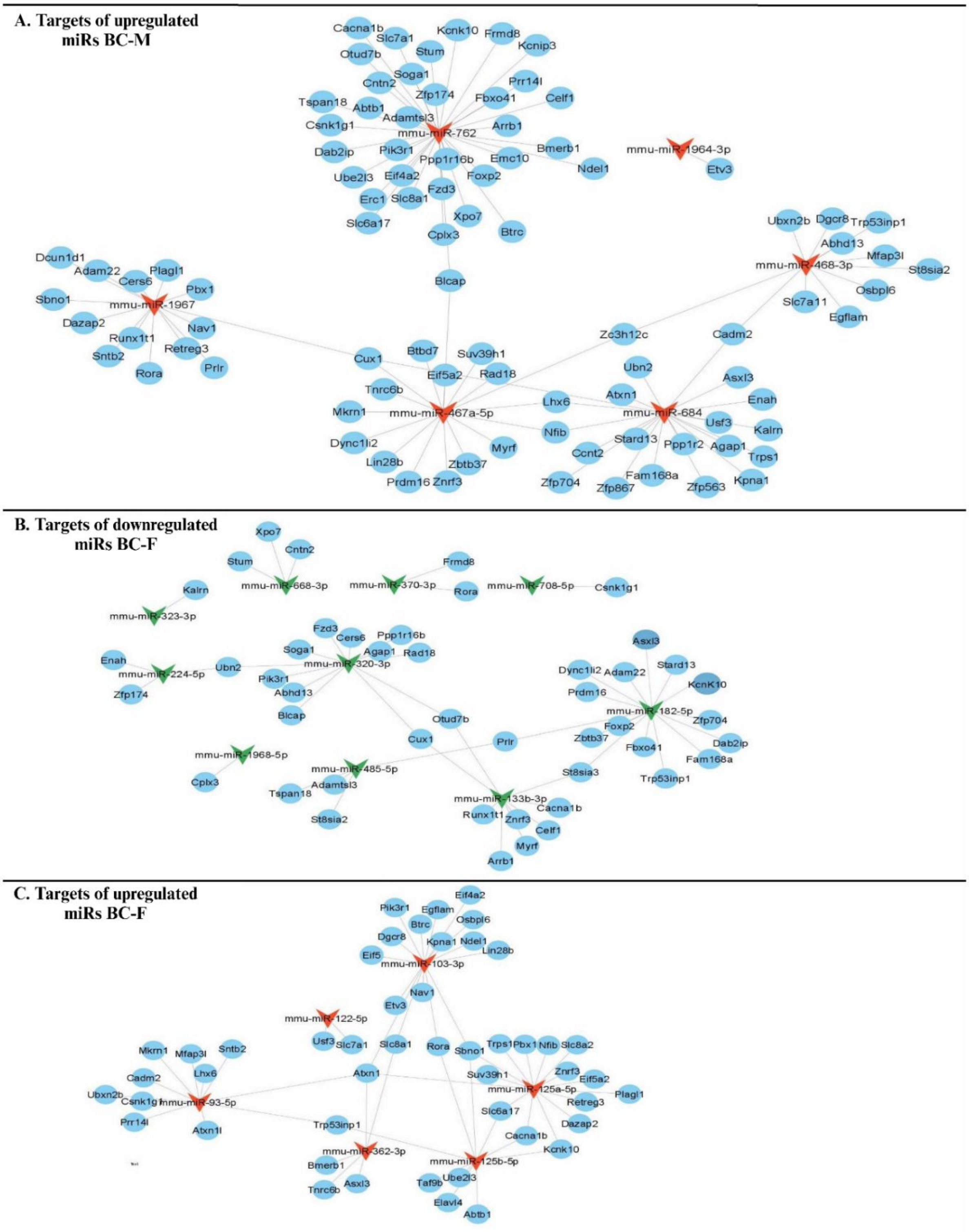

Figure S2. miRNA-gene interaction networks of the 86 overlapping targets across sexes of miRNAs differentially expressed with neonatal beta-carotene (BC) supplementation in the liver of mouse offspring born to Western diet-fed mothers and the 22 miRNAs potentially involved in their regulation. A) Targets of upregulated miRs in males; B) Targets of downregulated miRs in females; C) Targets of upregulated miRs in females. The single miR downregulated with BC in the liver of males was not predicted to interact with any of the 86 overlapping target genes across sexes.

International Journal of *Molecular Sciences*

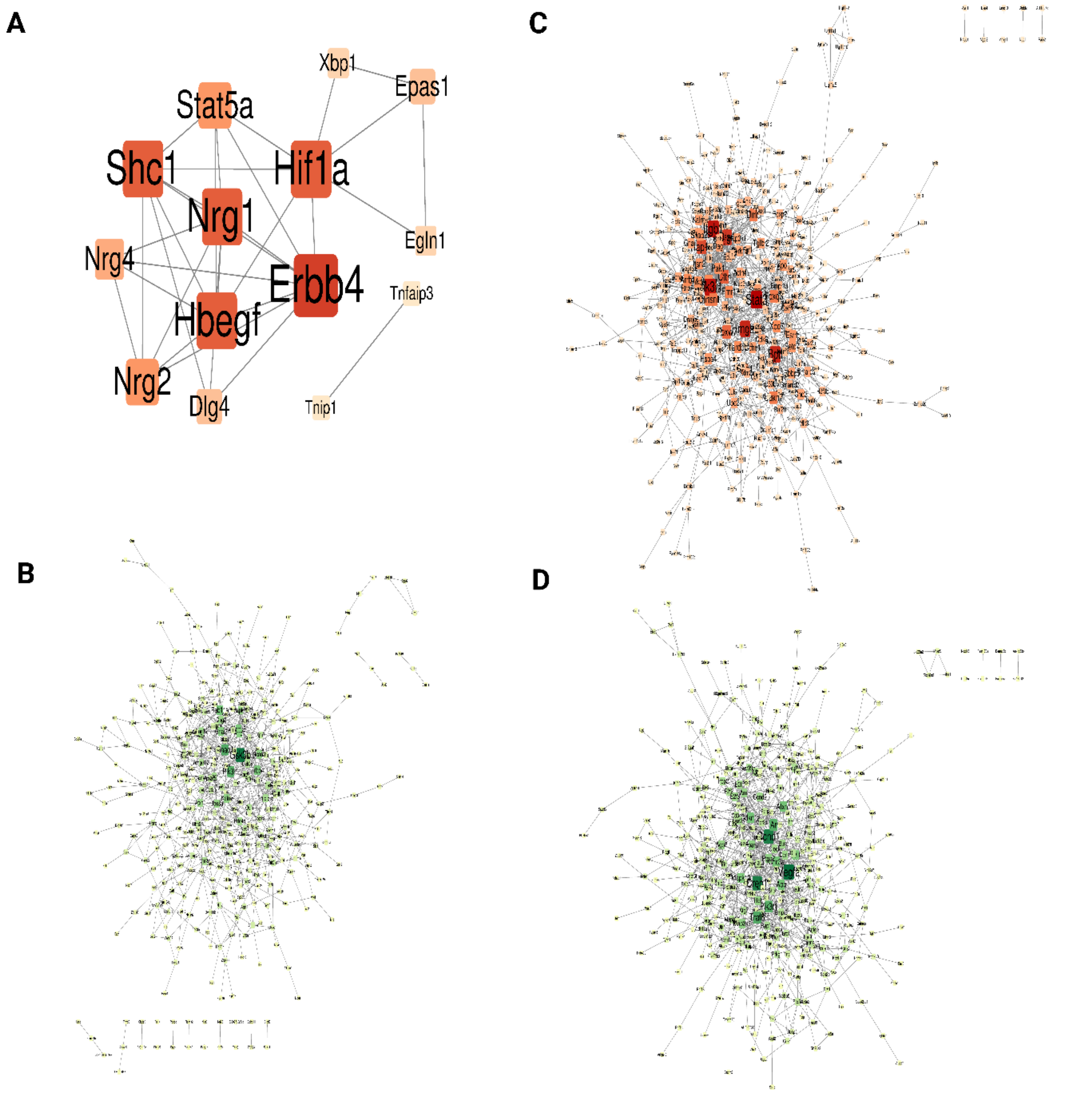

Figure S3. Protein-protein interaction (PPI) networks of predicted targets of the miRNAs differentially expressed with neonatal beta-carotene (BC) supplementation in the liver of offspring of Western diet-fed mouse dams. Each node is a protein, and an edge is an interaction between two proteins. **A**) PPI of the downregulated miRNA targets in BC males **B**) PPI of upregulated miRNA targets in BC males **C**) PPI of downregulated miRNA targets in BC females **D**) PPI of upregulated miRNA targets in BC females.
